# The rare and undiagnosed diseases diagnostic service – application of massively parallel sequencing in a state-wide clinical service

**DOI:** 10.1186/s13023-016-0462-7

**Published:** 2016-06-11

**Authors:** Gareth Baynam, Nicholas Pachter, Fiona McKenzie, Sharon Townshend, Jennie Slee, Cathy Kiraly-Borri, Anand Vasudevan, Anne Hawkins, Stephanie Broley, Lyn Schofield, Hedwig Verhoef, Caroline E. Walker, Caron Molster, Jenefer M. Blackwell, Sarra Jamieson, Dave Tang, Timo Lassmann, Kym Mina, John Beilby, Mark Davis, Nigel Laing, Lesley Murphy, Tarun Weeramanthri, Hugh Dawkins, Jack Goldblatt

**Affiliations:** Genetic Services of Western Australia, Department of Health, Government of Western Australia, Perth, WA Australia; School of Paediatrics and Child Health, University of Western Australia, Perth, WA Australia; Institute for Immunology and Infectious Diseases, Murdoch University, Perth, WA Australia; Office of Population Health Genomics, Public Health Division, Department of Health, Government of Western Australia, Perth, WA Australia; Telethon Kids Institute, University of Western Australia, Perth, WA Australia; Diagnostic Genomics, PathWest, Department of Health, Government of Western Australia, Perth, WA Australia; Centre for Population Health Research, Curtin Health Innovation Research Institute, Curtin University of Technology, Perth, WA Australia; School of Pathology and Laboratory Medicine, University of Western Australia, Perth, WA Australia; Centre for Comparative Genomics, Murdoch University, Perth, WA Australia; Public Health Division, Department of Health, Government of Western Australia, Perth, WA Australia; Western Australian Register of Developmental Anomalies, Perth, WA Australia; School of Spatial Sciences, Curtin University, Perth, WA Australia; Cooperative Research Centre for Spatial Information, Perth, WA Australia; Centre for Medical Research, Harry Perkins Institute of Medical Research, QEII Medical Centre, University of Western Australia, Perth, WA Australia; Rare Voices Australia, Sydney, Australia

**Keywords:** Diagnosis, Genomics, Undiagnosed, Diagnostic odyssey, Clinical best practice, Policy, Precision public health

## Abstract

**Background:**

The Rare and Undiagnosed Diseases Diagnostic Service (RUDDS) refers to a genomic diagnostic platform operating within the Western Australian Government clinical services delivered through Genetic Services of Western Australia (GSWA). GSWA has provided a state-wide service for clinical genetic care for 28 years and it serves a population of 2.5 million people across a geographical area of 2.5milion Km^2^. Within this context, GSWA has established a clinically integrated genomic diagnostic platform in partnership with other public health system managers and service providers, including but not limited to the Office of Population Health Genomics, Diagnostic Genomics (PathWest Laboratories) and with executive level support from the Department of Health. Herein we describe report presents the components of this service that are most relevant to the heterogeneity of paediatric clinical genetic care.

**Results:**

Briefly the platform : i) offers multiple options including non-genetic testing; monogenic and genomic (targeted in silico filtered and whole exome) analysis; and matchmaking; ii) is delivered in a patient-centric manner that is resonant with the patient journey, it has multiple points for entry, exit and re-entry to allow people access to information they can use, when they want to receive it; iii) is synchronous with precision phenotyping methods; iv) captures new knowledge, including multiple expert review; v) is integrated with current translational genomic research activities and best practice; and vi) is designed for flexibility for interactive generation of, and integration with, clinical research for diagnostics, community engagement, policy and models of care.

**Conclusion:**

The RUDDS has been established as part of routine clinical genetic services and is thus sustainable, equitably managed and seeks to translate new knowledge into efficient diagnostics and improved health for the whole community.

## Background

Rare diseases are a health priority that collectively are estimated to affect up to 6–8 % of the population [1–4]. Many rare diseases have their onset in childhood, continue lifelong, are complex with multisystem dysfunction and are disabling and burdensome to individuals, families and the healthcare system. There are 5,000–8,000 known rare diseases, and because of their individual rarity achieving a diagnosis is particularly challenging. In a European study, 25 % of individuals waited 5–30 years for a diagnosis and in 40 % of instances the initial diagnosis was wrong [5]. A recent survey showed similar findings in Australia including that approximately 30 % of patients waited for more than 5 years to receive a diagnosis, a similar number saw more than 6 doctors before receiving a diagnosis and half had at least one incorrect diagnosis [6] . Reflecting the priority for the global rare diseases community, local and international efforts have been developed to address this diagnostic odyssey as an accurate diagnosis is the bedrock of best practice medical care. Stated differently, the “Diagnosis—the Value of Knowing— is the foundation of quality healthcare. Its value is undeniable—to patients, to doctors, and to the underlying financial health of our healthcare system”. Furthermore, “accurate diagnosis—and the appropriate use of diagnostic tools—is a key driver toward the successful transformation of our healthcare system” [7].

Given that most rare diseases are genetic in origin, the diagnostic process historically often included a consultation with a clinical genetic service which consisted of a clinical assessment followed by investigations which may include sequential monogenic testing where deemed clinically appropriate. The advent of chromosomal microarray, followed by the clinical application of massively parallel sequencing, either targeted (in the sense of a multi-gene panel) or genome wide (whole exome or whole genome), has resulted in a markedly increased diagnostic yield in rare diseases [8] and this is modifying the diagnostic paradigm. International experience with the clinical implementation of genomic sequencing across the diversity of presentations typical of clinical genetic practice, showed a diagnostic yield around 25-30 %. Yang et al. reported an observational study of whole-exome sequencing which provided a molecular diagnosis for 25 % of a cohort of predominantly paediatric patients with suspected genetic disorders. The sub cohort with the greatest detection rate was for neurological plus other organ system involvement [9]. Retterer et al. described a diagnostic yield of 28.8 % for a heterogeneous group of presentations. The highest yield was for patients who had disorders involving hearing, vision, the skeletal muscle system, the skeletal system and multiple congenital anomalies [10]. Lee et al. reported the outcomes of consecutive patients with undiagnosed suspected genetic conditions seen at a single clinical genetic centre. Most cases were submitted from geneticists and had substantial prior genetic investigation. They often presented with clinical symptoms that either involved more than one body system or were deemed to be highly genetically heterogeneous. Approximately two thirds were children. The overall molecular diagnosis rate was 26 % [8]. Atwal et al. reviewed their institutions clinical experience and surveyed multiple other centres, from this heterogeneous group of centres and testing platforms, cumulatively there was 22.8 % success rate [11].

Higher success rates have occurred in the setting of research projects with highly selected cohorts, as distinct from the heterogeneity of presentations as seen in a clinical practice setting.

It is important to balance the diagnostic yield and the multiple attendant benefits of a confirmed molecular diagnosis (see Table 1) against the direct (test, analysis) and potential indirect costs of testing (such as with follow up of variants of uncertain significance). These considerations are especially important in the setting of limited health budgets and with consideration of *primum non nocere*. Formal health economic and econometric studies are underway to provide important evidence for helping to explore and evaluate financial questions. Whilst these are awaited, the burden on patients, their families and clinical service providers of the unmet clinical need for diagnosis in genetic and rare diseases demands a pragmatic and timely approach that can immediately deliver against this need, whilst providing clinical data to assist ongoing economic studies that are tailored to the local health context. Equally, it is crucial that service delivery is provided equitably. We describe the initial outcomes of the RUDDS, a diagnostic pipeline in a public health system operating from within a state-wide clinical genetic service to address the diagnostic odyssey of individuals living with rare diseases.

**Table 1:** The power of a diagnosis

Benefits	Comments
Certainty	The power of knowing the cause of the condition at the end of the diagnostic odyssey, including improved prognostication.
Reduced Isolation	Offering the possibility of connection for shared experience.
Reduce unnecessary investigations	No further need for investigations which may be invasive, time-consuming and/or costly.
Access to improved or best practice medical care, including reducing inappropriate management	Targeted follow-up and surveillance by what is known from the diagnosed condition and biologically related disorders. Possibility of drug repurposing^a^.
Clarify recurrence risk	To increase certainty and restore reproductive confidence.
Provide additional reproductive options	A molecularly confirmed genetic diagnosis provides options for prenatal or pre-implantation genetic diagnosis.
Access to social and educational services	Available for selected other rare disorders.

^a^drug repurposing: using a given drug for a new indication (disease)

## Methods

**Fig. 1:**
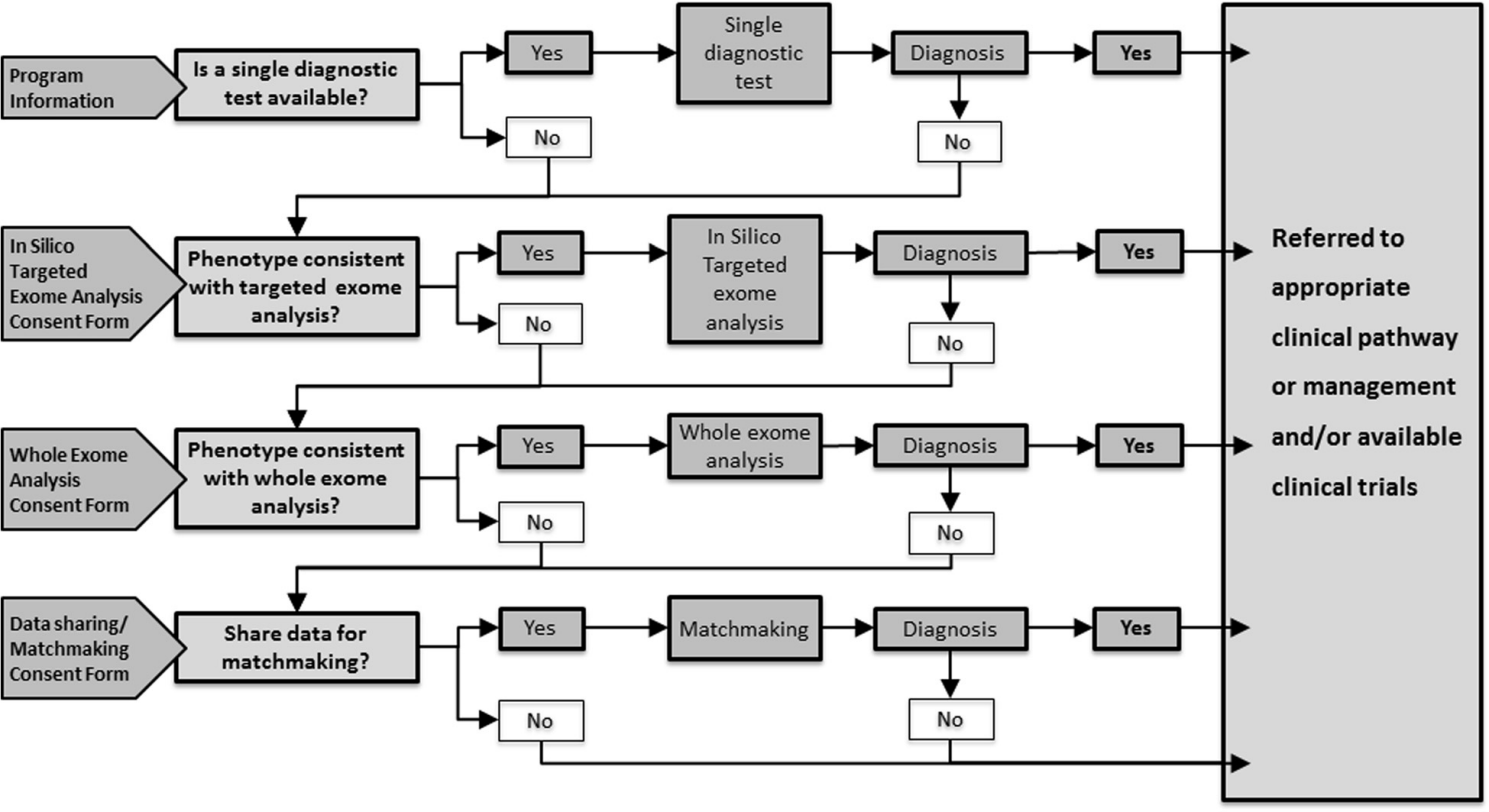
Program schematic. This schematic represents the Rare and Undiagnosed Diseases Diagnostic Service (RUDDS)

Individuals seen in outpatient clinics at GSWA received a genetic consultation and clinical assessment (phenotyping) by the attending physician. This includes: ascertainment of history; physical examination, including 2-dimensional and 3-dimensional photographs, anthropometric measurements; and documentation of relevant investigation results. Relevant complex undiagnosed cases, included, but were not limited to, when in silico filtered or whole exome/ genome analysis was being considered. Cases were presented at a weekly multi-clinician meeting to achieve a consensus for further investigations such as (i) non-gene-based testing (e.g. imaging and biochemistry), to either confirm a diagnosis or to refine the phenotype prior to, or in partnership with, genetic testing; (ii) recommendation of additional expert review; (iii) monogenic testing; and (iv) targeted in silico filtered exome or whole exome analysis. A clinical consensus decision directed investigation course e.g. request for further phenotyping, including deferring of further testing with review with the passage of time, or the variety of testing options, including genomic ones. Of those that preceded to genomic testing, the majority (74/77) analyses were performed as in silico filtered panels, with familial follow-up of variants as required. The approach to whole exome sequencing (e.g. sib-pair, trio or most distantly related family members with the same unique phenotype) was a case-by-case decision made with discussion between clinical and laboratory staff. For those where massively parallel sequencing failed to identify a pathogenic mutation, a matchmaking service for undiagnosed patients was offered. Specifically, in the first instance, by offer of submission to Phenome Central. Additionally, patients were offered recruitment into research projects aimed at identifying a diagnosis through the development of novel genomic analyses, principally SeqNextGen which is a translational research program contributing to improving the diagnosis of rare genetic diseases and models of care for genomic diagnostics for rare diseases and developmental anomalies. SeqNextGen was implemented as a recipient of a pipeline from the RUDDS with aims including to improve data analytics for rare diseases diagnosis, and relatedly to be adaptable to the heterogeneity and temporal evolution of in-service clinical genomic testing. Those without (and with) a confirmed genomic diagnosis are given the option to share genomic data with this study. Those with a confirmed genomic diagnosis serve as positive controls for validation of new data analytic approaches. The data from those without a diagnosis can be re-analysed with new approaches in case this yields a definitive answer. Any potential diagnosis is then discussed with managing clinician before any clinical laboratory confirmation is performed prior to contacting the patient. This project also incorporates patient and family experience surveys. A schematic of the RUDDS is in Fig. 1.

The design of in silico filtered panels was performed as a partnership between the laboratory staff and clinicians and pragmatic decisions balancing cost, throughput, detection rate, available monogenic testing, laboratory and clinical experience of the phenotype under investigation, and the likely rate of determining variants of uncertain significance were made. Largely, gene panels were created to cover groups of presentations that are frequently represented in clinical genetics clinics, including multisystem diseases, with and without intellectual disability, that are typically associated with locus heterogeneity and often involve common biological pathways. Panels were iterated from a foundation of more than 200 in-house monogenic tests. They included tests for cohesinopathies and phenotypically/biologically related disorders (e.g. Cornelia de Lange Syndrome and BAF complex disorders (e.g. Coffin-Siris syndrome)) disorders associated with absent or severe speech impediment (e.g. Angelman syndrome and overlapping disorders); RASopathies (e.g. Noonan syndrome); overgrowth disorders (e.g. Sotos syndrome); ciliopathies (e.g. primary ciliary dyskinesias and autosomal recessive polycystic kidney disorders); and presumptive X-linked mental retardation. Less frequently, other selected disorders were investigated based on clinical need coupled with locus heterogeneity and/or on prohibitive costs of send-away tests. Creation of in silico targeted panels that was performed for such purposes included those for Kabuki syndrome, epidermolysis bullosa, cutis laxa-associated conditions, corneal dystrophies and hereditary haemorrhagic telangiectasia.

Reflecting the practicality of testing within a clinical setting and the rapidly evolving technological landscape, massively parallel sequencing was performed on multiple platforms. Briefly, libraries were prepared using the Ion Ampliseq Exome Kit (Life Technologies) or with the NEBNext Ultra DNA Library Kit (New England BioLabs) and captured with the TargetSeq Exome Kit (ThermoFisher Scientific). Sequencing was performed on the Ion Proton Sequencer (Life Technologies) or the SOLiD 5500XL (Applied Biosystems) instruments, respectively. A minimum of 85 % of gene regions included in the data analysis were covered to a depth of at least 20 fold; typically the proportion was much higher (>90 fold) for most samples. Alignment and variant calling were performed using Torrent Suite software (Life Technologies) or LifeScope software (Applied Biosystems). Variants were annotated, filtered and analysed in Bench Lab NGS (Cartagenia).

Known or likely pathogenic variants were confirmed by Sanger sequencing in a National Association of Testing Laboratories, Australia (NATA)-accredited laboratory (Diagnostic Genomics, PathWest).

Close liaison between clinicians, genetic pathologists and other diagnostic genomic scientists was frequent and facilitated by regular meetings. Genetic counsellors provided critical expertise to all parts of the diagnostic platform including patient consent, coordination and ongoing patient and family support. The clinical service and associated translational research was run in partnership with the key input of patient advocacy groups, including Rare Voices Australia (www.rarevoices.org.au australia.org.au).

Importantly, equitable health care delivery was supported by delivering the RUDDS within a clinical state-wide public health service that is a focused on improving genetic health care for the community in the metropolitan area and, through outreach pathways, to rural and remote regions providing, including Aboriginal and non-Aboriginal Australians.

Herein, we focus primarily on children with the heterogeneous presentations typical of paediatric clinical genetics who were assessed through the diagnostic platform and progressed to in-house genomic investigations (in silico targeted panels, whole exome sequencing) from July 2013 – July 2015. We have excluded neuromuscular diseases which are largely managed through neurological services; cardiac diseases (structural, electrical and aortopathies) and familial cancers which in the main have been provided for adults through familial cancer or cardiogenetic pathways; and all genomic investigations performed out of state or country. We also excluded those diagnosed by epigenetic, radiological or other means.

## Results and discussion

The overarching achievement is the creation of an imperfect but agile and iteratively improving, diagnostic platform within the public health service, that is aligned to unmet need, and that supports equitable state-wide diagnostic health care provision through the integration of genomic diagnostics. Notably, this improved in-house causative monogenic detection rate from a historical service baseline of 10 – 30 % for this heterogeneous and diagnostically challenging clinical cohort. The RUDDS is consistent with fundamental tenants of clinical genetic service delivery, including excellent phenotyping, partnership with genetic pathologists and laboratory staff and alignment to the patient journey. Primary outcomes of the RUDDS included:

Firstly, that it was delivered in a patient-centric manner resonant with the patient journey, specifically that as it is not limited to the duration of a research project, nor to a static consent process, that patients can engage with or leave the platform at various stages and at times relevant to them, providing news they can use and when they are ready to receive it. Additionally, through provision in a state-wide service, including outreach clinics, it could be provided with geographic equity. Secondly, by integration in clinical service , multiple options including monogenic and genomic (targeted and whole exome) analysis, non-gene based testing, and matchmaking could be offered. Thirdly, it was synchronous with precision phenotyping methods, including that 3-D facial analysis could be provided. Even if it was “just” the provision of a 3-D image that could be interrogated at clinical case review to provide an increased depth of information compared to 2D images. Fourthly, it captured new knowledge, including multiple expert reviews, specifically the documentation and process of team discussions around each individual either before and/ or after testing provided opportunities for collective up-skilling with likely future benefit to future families. For instance, multiple cases with clinical indicators of possible mosaicism and normal testing on peripheral blood, altered clinical practice for tissue selection in subsequent cases, and lead to new processes for acquiring the relevant samples. Additionally, the new knowledge of the *mTOR* mutation offered a new alternative for seizure treatment. Fifthly, integration with current translational genomic research activities, specifically, but not limited to the the SeqNextGen project described above. In some instances, patients that could not be resolved with the SeqNextGen project were recruited into research projects with laboratories with specific phenotype domain expertise. Also, as a by-product of the relationships established through this collaboration, and following the case of a familial *mTOR* described above, the first genomic reference range data for Aboriginal Australians [12] was translated from research to implementation as de-identified frequency data in our clinical laboratory; Sixthly) flexibility for integration with further clinical research including additional diagnostic approaches, knowledge management platforms, community engagement, policy and models of care. This includes: i) whole genome sequencing; ii) the planned implementation of a knowledge management platforms for mining of free text, case sharing, discussion and an application programming interface to push data to Matchmaker Exchange, namely Patient Archive; iii) community engagement, such as that enabled through the delineation of a critical mass of diagnosed and undiagnosed patients and relatedly by directly partnering with community peak bodies to develop policy and models of care. Finally, less tangible outcomes included: increased workplace satisfaction for staff though improved access to molecular confirmation, as indicated by repetitive informal feedback, and noting that this was not assessed through formal surveys during the period described; and the potential for professional development through the shared experiences and quality improvements resulting from definitive diagnoses.

The overall mutation detection rate was in accordance with expectation at 30 %. Similar to other reports [8–10], the diagnostic yield was proportional to the specificity of the described phenotype, including affectation of multiple organ systems, rather than a single organ system. This was reflected in the following mutation detection rates hereditary haemorrhagic telangiectasiae (5/5, 100 %), RASopathies and overgrowth disorders (7/16, 44 %) and presumed X-linked mental retardation (0/7, 0 %).

Two illustrative cases are described. Firstly, over a 10 year period an Aboriginal family from remote Australia had been seen in an outreach clinic. Multiple siblings had the same unique phenotype characterised by intellectual disability, macrocephaly, small thoraces, asymmetric visceral overgrowth, connective tissue dysplasia, predisposition to infection, and variably one to multiple cafe-au-lait lesions, hemi-megalencephaly with perisylvian polymicrogyria. Over the decade, multiple diagnoses had been considered and many monogenic tests had been performed with normal results, largely to investigate RASopathies and overgrowth disorders. Guided by the phenotype, WES was targeted to genes known to be associated with the two presumptive diagnostic groups and overlapping genes, including *mTOR*. A likely pathogenic variant in *mTOR* was identified. The mutation segregated with disease in the family. Following a protracted search for a suitable laboratory, functional studies were performed that supported a gain of function mechanism for this gene [13]. This finding also supported the possibility of mTOR inhibitor drug repurposing. This case highlights the importance of phenotyping; the need for coordinated access to functional studies, such as in the first instance might be enabled by a virtual network of functional analyses; and management implications of a molecularly confirmed diagnosis.

In the second example, a child presented with features consistent with megalencephaly-capillary malformation syndrome; specifically, prenatal onset impressive macrocephaly, infantile onset hydrocephalus, capillary malformations and connective tissue dysplasia. Targeted exome sequencing of *PIK3CA* and related genes was performed on DNA extracted from peripheral blood and skin fibroblasts; a mosaic mutation was detected in the latter but not the former and was subsequently confirmed by Sanger sequencing of DNA from skin fibroblasts and cerebrospinal fluid. This case again highlights the importance of phenotyping to guide genomic analysis and highlights the selection of an appropriate tissue for testing.

Provision of this service within a public health setting and with multi-expert review was an approach that was tailored to local circumstances, including optimal use of limited health resources targeted to the unmet need of a population where it is was most likely to have immediate clinical utility and deliver patient benefits. Key to the patient-centric delivery of this service was the pivotal involvement of genetic counsellors at multiple points in the diagnostic pipeline and for ongoing support as required. Other critical success factors included partnerships with translational research to augment future diagnostic analytic capacity, promotion of access to functional analysis, and the initiation of economic analyses and studies of patient and family experience, as well as the development of improved models of care. Similarly, active community engagement was informative and supportive at all stages. Finally, the involvement of a public health genomic policy unit supported implementation and sustainability.

By iterating within the clinical service, known or unanticipated real-world bottlenecks can be identified and pragmatically addressed. Also, real-time in-service studies of health care provision can be performed and are in progress. Process improvements to address identified bottlenecks will need to include more timely ascertainment of human phenotype ontology terms in a manner aligned to clinical flow [14], as this will streamline communications with the clinical laboratory and facilitate the better use of phenotypic information in the analytic workflow. Also, the need for capacity building including promoting the development of a workforce that is knowledgeable of the evolving relative place and limitations of genomic and non-genomic investigations. Additionally, given the significant proportion of cases that remained undiagnosed, the need for complementary approaches for diagnostically intractable cases, including local and international undiagnosed diseases programs [15], was reinforced.

A limitation of this work which might also be viewed as a critical success factor, was the lack of internal consistency of some technical instrumentation and analytic tools used during the evaluation of this cohort. This was reflective of and responsive to changes in technology and was a testament to the endeavour and flexibility of the laboratory team that delivered within the heterogeneity and imperfection of clinical process. What was also demonstrated very clearly was that it was the phenotype-informed and patient-centred processes that were instrumental to applying technology to enable improved diagnostic yield.

## Conclusion

We describe an iteratively improving diagnostic platform provided within a public health service that is aligned to the unmet needs of people living with rare and undiagnosed diseases, by supporting equitable state-wide diagnostic health care provision for the world’s geographically largest public health jurisdiction. It is largely been performed within existing budgets through a patient-centric approach and clinically informed re-alignment of existing resources.

## Abbreviations

GSWA, Genetic Services of Western Australia; RUDDS, Rare and Undiagnosed Diseases Diagnostic Service; WES, whole exome sequencing
